# Caregiver Burden and Associated Factors for the Respite Care Needs among the Family Caregivers of Community Dwelling Senior Citizens in Chiang Mai, Northern Thailand

**DOI:** 10.3390/ijerph18115873

**Published:** 2021-05-30

**Authors:** Thin Nyein Nyein Aung, Myo Nyein Aung, Saiyud Moolphate, Yuka Koyanagi, Siripen Supakankunti, Motoyuki Yuasa

**Affiliations:** 1 Department of Public Health, Graduate School of Medicine, Juntendo University, Tokyo 113-8421, Japan; a-thin@juntendo.ac.jp (T.N.N.A.); moyuasa@juntendo.ac.jp (M.Y.); 2 Advanced Research Institute for Health Sciences, Juntendo University, Bunkyo City, Hongo, 2 Chome-1-1, Tokyo 113-8421, Japan; 3 Faculty of International Liberal Arts, Juntendo University, Tokyo 113-8421, Japan; 4 Department of Public Health, Faculty of Science and Technology, Chiang Mai Rajabhat University, Chiang Mai 50300, Thailand; saiyudmoolphate@gmail.com; 5 Tokyo Ariake University of Medical and Health Sciences, Tokyo 135-0063, Japan; koyanagiy@tau.ac.jp; 6 Centre of Excellence for Health Economics, Faculty of Economics, Chulalongkorn University, Bangkok 10330, Thailand; Siripen.S@chula.ac.th

**Keywords:** aging, Asia, community integrated intermediary care (CIIC), Caregiver Burden Inventory (CBI), long-term care, respite care

## Abstract

Background: Families are the backbone of caregiving for older adults living in communities. This is a tradition common to Thailand and many low- and middle-income countries where formal long-term care services are not so available or accessible. Therefore, population aging demands more and more young people engaging as family caregivers. Informal caregiving can become an unexpected duty for anyone anytime. However, studies measuring the burden of informal caregivers are limited. We aimed to determine the caregiver burden, both from the perspective of the caregivers as well as that of their care recipients. Method: We used the baseline survey data from a cluster randomized controlled trial providing a community integrated intermediary care (CIIC) service for seniors in Chiang Mai, Thailand, TCTR20190412004. Study participants were 867 pairs of older adults and their primary family caregivers. Descriptive analysis explored the characteristics of the caregivers and binary logistic regression identified factors influencing the caregivers’ burden. Results: The mean age of family caregivers was 55.27 ± 13.7 years and 5.5% indicated the need for respite care with Caregiver Burden Inventory (CBI) scores ≥24. The highest burden was noted in the time-dependence burden domain (25.7%). The significant associated factors affecting CBI ≥24 were as follows: caregivers older than 60 years, being female, current smokers, having diabetes, and caring for seniors with probable depression and moderate to severe dependency. Conclusions: A quarter of caregivers can have their careers disturbed because of the time consumed with caregiving. Policies to assist families and interventions, such as respite service, care capacity building, official leave for caregiving, etc., may reduce the burden of families struggling with informal care chores. Furthermore, caregiver burden measurements can be applied as a screening tool to assess long-term care needs, complementing the dependency assessment. Finally, implementation research is required to determine the effectiveness of respite care services for older people in Thailand.

## 1. Introduction

Population aging is happening worldwide, and by 2050, there will be an estimated population of 2.1 billion older people globally [1]. In the midst of this global phenomenon, Thailand is experiencing one of the most rapid rates of population aging in the developing world, with the older population expecting to increase to 20 million, accounting for 35.8% of the population [2,3]. In addition, Thailand is currently home to the second highest population of older people in Southeast Asian countries, second only to Singapore [4].

Family is the traditional social institution for the care of older adults globally, and likewise in Thailand, older people mainly rely on their family for health and material support. Cultural and social norms stipulate that family members provide support to older persons in the form of co-residence, with these caregivers acting through feelings of gratitude, influenced by Buddhism, the national religion of Thailand [5,6]. According to Thai culture, a sense of obligation or filial duty is very important for Thai families, whereby caregiving is regarded as a generalized reciprocity, with caregivers giving back to their parents who provided care for them when they were children. As a result, in Thailand and much of Asia, the family, particularly adult children, have traditionally played the predominant role in providing old age care and support [7,8,9]. However, this family-based long-term care is challenged by demographic changes, particularly in terms of population aging, increased life longevity, the reduction in family size, and the migration of adult children for their job opportunities [10].

Caregiving by family members is a form of informal care, whereby family caregivers provide care to older adults in their families. Population aging demands more and more young people engaging as family caregivers, and informal caregiving can become an unexpected duty for anyone anytime. The caregiver burden is a state resulting from the care of older care recipients, threatening the physical or psychological well-being of the caregiver. It is defined as “a multidimensional response to physical, psychological, emotional, social, and financial stressors associated with the caregiving experience” [11]. The perception of burden will determine the impact of caregiving on the caregiver’s life, and while some caregivers may find it rewarding, others may find it challenging and burdensome. Caregivers, busy taking care of others, can often forget about their own health. Caregiving demands can easily overwhelm caregivers’ physical and mental health, which can lead to fatigue, hopelessness, and ultimately, burnout. Caregiver burnout is a progression of burden to the point where the experience is no longer a healthy option for either the caregiver or the care recipient. When the caregiving burden escalates to such an extent, this not only affects the health of the caregiver, but also their care recipients and the rest of the family too.

A community integrated intermediary care service model, via a cluster randomized controlled trial (CIIC study), was introduced to address these issues. This represents a new service model, consisting of three components: care prevention exercise, family caregiver training, and intermediary care service. Care prevention exercise, in the form of a functional training exercise program, is delivered to ambulatory older people. For families with dependent older adults, family caregiver training is provided through technical support and advice for caregiving depending on their needs. The intermediary care service is a free of charge, formal care, short-stay service at the local community CIIC center, staffed by health care personnel. This respite care service aims to relieve Thai families of the caregiving burden by taking care of older people when their caregiver experiences symptoms of burnout or is temporarily unavailable [12].

Research shows that the provision of such respite care services can help caregivers take a break from caregiving and also prevent the likelihood of abuse and neglect of the elderly care recipient [13,14,15]. Providing assistance to family caregivers is crucial, as this could help reduce the need for institutionalization or hospitalization of the elderly person, and subsequently, lower the expenditure on long-term care at household and national levels. Furthermore, with rising life expectancies, these current family caregivers will soon become eventual older care recipients themselves, meaning a sustainable family-based long-term care model for the Thai community should aim towards active and healthy aging of both care recipients and their caregivers. Therefore, we aimed to assess the extent of the family caregiver burden and determine the extent of burnout and the needs for respite care. The determinants of high caregiver burden were explored from the aspects of the care recipients’ demands and the family caregivers’ characteristics, using the baseline data from the intervention arm of the CIIC study. The findings of this research may be useful in estimating the service demands for a CIIC short-stay service in relation to the caregiver burden among family caregivers of community-dwelling older adults in the setting of Northern Thailand.

## 2. Materials and Methods

### 2.1. Data Collection and Participants

This study was conducted in accordance with the Declaration of Helsinki. The World Health Organization Ethical Review Committee: WHO/ERC ID; ERC.0003064, dated 7 March 2019 and the Ethical Review Committee for Research in Human Subjects: Boromarajonani College of Nursing Nakhon Lampang: Praboromarajchnok, Institute for Health Workforce Development, Ministry of Public Health, Thailand (approval number E 2562/005, dated 4 March 2019) approved the ethics of the study. It was registered at the Thailand Clinical Trial Registry—Trial registration number TCTR20190412004.

This study was part of a separate cluster randomized controlled trial: a community integrated intermediary care (CIIC) project, consisting of six intervention and control clusters, and comprising 2000 participants in each arm. Data were taken from the baseline survey of one intervention arm of this study, prior to launch of the intervention. STATA version 11SE (Stata Corporation, College Station, TX, USA) was used for sample size calculation and power estimations. Inclusion criteria were care recipients aged ≥60 years and their family caregivers, either male or female, who were taking care of them at home, and being a resident in the study site for at least 1 year. Those who were unable to understand about informed consent, who had cognitive impairment, or who did not consent, were excluded. Following CIIC study protocol, data were collected by trained research assistants via interviewer administered survey questionnaires from August to December 2019 [12]. Maehia city, Chiang Mai province, Northern Thailand was randomly selected as the intervention arm and 1509 older people, and 867 primary caregivers were recruited with their written informed consent. All primary caregivers and their respective care recipients were selected to be included in this subgroup analysis and, therefore, a total of 867 pairs of Thai seniors and their family caregivers were included in this study.

### 2.2. Procedures of Cluster Randomized Control Trial

The cluster randomized control trial (CIIC project) consisted of 6 intervention clusters and 6 control clusters. The control arms received routine elder care services provided by the municipality, i.e., the current system of long-term care common to all provinces in Thailand, which included a volunteer-assisted home care service. The intervention clusters were introduced to a new service model that consisted of three components: (1) care prevention exercise for ambulatory older persons, (2) family caregiver training for family caregivers of dependent older adult care recipients, and (3) an intermediary care service at the CIIC center. A short-stay service for the older adults aimed to provide a temporary break for their caregivers when they felt overburdened or were temporarily unavailable. The CIIC center was staffed by health care personnel, and provided a free of charge, full-time formal care service for the older adults for 10–14 days. The families who registered for a short-stay service at the CIIC center were screened for eligibility using Barthel’s Activity of Daily Living scores (ADL scores) in relation to the care recipients’ demands, and the Caregiver Burden Inventory (CBI scores) in relation to the caregivers’ burden. According to the dependency of the care recipient and the caregiver’s burden, ambulatory elderly participants and non-burdened caregivers received a care prevention exercise program, whilst burdened caregivers and caregivers of dependent care recipients were provided with care capacity-building educational programs and a respite care service at the CIIC center.

### 2.3. Measures

Interviews were conducted with the study participants using a structured questionnaire. It contained three parts: (1) sociodemographic characteristics, (2) assessment of the caregiver burden of the family caregivers, and (3) the care recipients’ demands. Sociodemographic characteristics of the family caregivers included the following variables: age, gender, education, marital status, occupation, being the main income supporter of the family, and the relationship with the care recipient. Health status was assessed according to underlying diseases such as hypertension, diabetes, and hyperlipidemia, and health behaviors like smoking, drinking alcohol, and exercise habits. The assessment of the level of family caregiver burden was calculated using the Caregiver Burden Inventory (CBI) score. Care recipients’ demands were assessed using Barthel’s index of Activities of Daily Living (ADL) and the 15-item Geriatric Depression Scale (GDS).

#### 2.3.1. Caregiver Burden Inventory (CBI)

This is an internationally validated measurement tool comprising a 24-item multi-dimensional questionnaire measuring the impact of burden on different aspects of a caregiver’s life, reflecting various areas of the caregiver’s well-being and function. It consists of 5 subscales: (a) time-dependence burden, which gives a measure of flexibility with time and the caregiver’s time restriction; (b) developmental burden, which evaluates the impact of failing to take opportunities and pursue goals; (c) physical burden, a measure of the physical consequences of caregiving (e.g., fatigue, bodily ache, and pain); (d) social burden, which assesses the impact on interpersonal and social relationships with the family and friends; and (e) emotional burden, which evaluates feelings of shame and embarrassment with respect to the care recipients. A 5-point Likert scale, ranging from 0 (not at all disruptive) to 4 (very disruptive) was used to evaluate each subscale [16]. All of the scores on the 24-item scale were added and categorized into two groups: (1) a total score of less than 24 group; and (2) a group of scores more than or equal to 24, indicating a need to seek some form of respite care.

#### 2.3.2. Barthel’s Activities of Daily Living (ADL) Index

This is a standardized scale widely used by clinicians and researchers to assess a person’s current level of ability to carry out daily activities. It consists of 10 fundamental items of daily living: feeding, grooming, bathing, dressing, bowel and bladder care, toilet use, ambulation, transfers, and stair climbing [17]. The total score ranges from 0 to 20 and is categorized into two groups: (1) a score of <12 indicates moderate to severe dependency, and (2) a score of ≥12 indicates mild dependency to independency.

#### 2.3.3. Geriatric Depression S Cale (GDS)

The 15-item Geriatric Depression Scale is commonly used internationally and has been employed as an effective depression screening tool within the Thai geriatric population. The Yes or No responses are scored according to scoring instructions and the total scores are dichotomized into “probable depression” (6–15) and “normal” (0–5) [18,19,20].

According to the WHO process of translation and adaptation of instruments, all of the study instruments were translated into Thai, back translated into English, and retranslated into Thai by independent language experts, as well as being reviewed and edited by researchers after pilot testing [21]. Cronbach’s alpha reliability coefficients of the CBI, ADL, and GDS were 0.77, 0.90, and 0.80 respectively.

### 2.4. Data Analysis

IBM SPSS version 22 (IBM Corporation, Armonk, NY, USA) was used for data analysis. Initially, the data were cleaned, and then an exploratory analysis was conducted (including recoding the variables and computing the subscales and scales as needed). Sociodemographic characteristics were analyzed by descriptive analysis. Frequency and percentages were used for the categorical variables (gender, education, marital status, occupation, and health behaviors—smoking, alcohol drinking and exercise habits, as well as the underlying diseases—hypertension, diabetes mellitus, and hyperlipidemia), and the mean (M) and standard deviation (SD) were used for the continuous variable (age). Binary logistic regression was applied to determine the factors associated with the need for respite care according to the CBI total scores ≥24. Binary logistics regression analysis was also carried out to explore the determinants of the caregivers’ time-dependence burden. An adjusted odds ratio (adj OR) with a 95% confidence interval and a *p* value of <0.05 was considered to be significantly associated.

## 3. Results

### 3.1. Sample Characteristics

**Figure 1:**
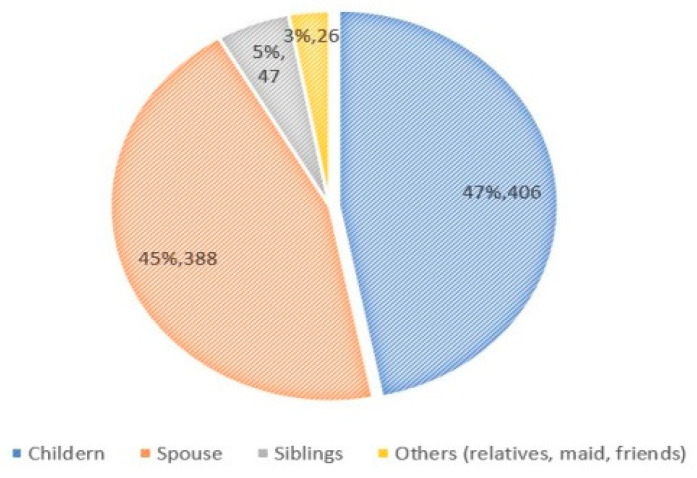
Relationship of primary caregivers with senior citizens, Maehia subdistrict, Chiang Mai, Thailand, 2019.

The findings were part of the baseline survey of a cluster randomized controlled trial, from the intervention arms comprising a total of 867 pairs of senior citizens and their primary family caregivers. The mean ages of the elderly care recipients and the family caregivers were 69.16 ± 8.33 years and 55.27 ± 13.7 years, respectively. Just over half of the caregivers were younger than 60 years of age (53.5%), more than one-third were 60–69 years old (34.9%), nearly 10% were 70–79 years (9.5%), and 2.1% were older than 80 years. The distribution of gender showed more female than male, both among care recipients and caregivers (57.7% vs. 42.3% and 62.3% vs. 37.7%, respectively). Almost two-thirds of the care recipients were married (64.5%). The children of care recipients contributed 46.8% of the family caregivers, followed by spouses (44.8%), siblings (5.4%), and other relatives, maids, and friends (3.0%), as shown in Figure 1. The median monthly income of family caregivers was about 9000 baht, and half (49.9%) were the main income supporter of the family, whilst 31.1% did not have a current job. About one-quarter (23.3%) of family caregivers were working at a government office/company and amongst them, 4.6% needed to take leave from their jobs frequently to look after their care recipients. The maximum frequency of leave was about 20 times in the previous year. Regarding health status, hypertension was the most prevalent underlying disease both for care recipients (46.3%) and family caregivers (28.0%), followed by hyperlipidemia (19.5%) and diabetes (17.4%) among care recipients, and diabetes (9.3%) and hyperlipidemia (8.2%) among family caregivers. When exploring the health behaviors of the care recipients and their caregivers, no exercise was reported by 26.4% and 21.0%, respectively, whilst drinking alcohol (22.7% vs. 27.7%) and currently smoking (6.5% vs. 8.8%) were also reported. Characteristics of the study participants are summarized in Table 1.

### 3.2. Family Caregiver Burden

**Figure 2:**
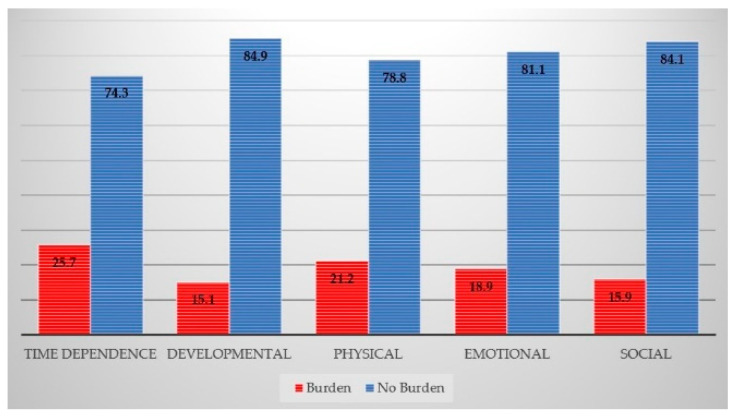
The distribution of family caregiver burden over the five domains considered in the Caregiver Burden Inventory, Maehia subdistrict, Chiang Mai, Thailand, 2019.

The family caregiver burden was calculated by adding up the total score of each Caregiver Burden Inventory (CBI) subscale (time-dependence, physical, emotional, social, and developmental). The burden in the time-dependence burden domain was highest (25.7%), followed by physical burden (21.2%), emotional burden (18.9%), social burden (15.9%), and developmental burden, (15.1%), as shown in Figure 2. The mean CBI total score was 4.41 ± 9.18 and 5.5% of family caregivers had a CBI total score ≥24, indicating the need for respite care to alleviate their caregiving burden.

### 3.3. Care Recipients’ Demands by ADL Total Scores

The mean age of the elderly care recipients was 69.16 ± 8.33 years, whilst the mean ADL total score was 19.08 ± 3.00. Almost all (96.7%) were mildly dependent to independent (ADL total scores more than or equal to 12), whilst 3.3% had moderate to severe dependency (ADL total scores less than 12). When exploring the details of ADL scores, the most common dependency in the activities of daily living was for bladder control (12.1%), followed by using stairs (8.1%), transfer (7.0%), mobility (7.0%), bowel control (6.5%), toilet use (5.5%), dressing (4.7%), feeding (4.4%), bathing (4.3%), and grooming (3.0%).

### 3.4. Care Recipients’ Demands by GDS Total Scores

About 6.6% of older adults had GDS total scores of 6–15, indicating that they had probable depression, whilst the remaining 93.4% did not have depression.

The summary of the care recipients’ demands, in terms of total ADL and GDS scores, and the family caregiver burden using CBI total scores, are shown in Table 2 below.

### 3.5. Factors Affecting the Family Caregiver Burden, Indicating the Needs for Respite Care

The family caregivers aged ≥60 years had caregiver burden scores 2 times higher than those younger than 60 years (adjusted odds ratio (Adj OR): 2.14, 95% CI: 1.03–4.44). There was a significant association between female family caregivers and their caregiving burden. The caregiver burden was about 2.3 times higher for female caregivers compared to male caregivers (Adj OR: 2.25, 95% CI: 1.02–4.94). Regarding the health status of family caregivers according to underlying diseases, the prevalence of having diabetes was 6.8 times higher than those not having diabetes (Adj OR: 6.76, CI: 2.75–16.59). There was no significant association between having hypertension or hyperlipidemia and a higher caregiver burden. Family caregivers who were current smokers had a caregiver burden nearly 5 times higher than non-smokers (Adj OR: 4.31, CI: 1.30–14.28). Even though most of the care recipients were mildly dependent in terms of total ADL scores, the caregiver burden of caregivers taking care of moderately to severely dependent care recipients was 6.4 times higher compared to those taking care of mildly dependent to independent care recipients (Adj OR: 6.36, CI: 2.37–17.06). It was noted that the caregiver burden was nearly 7 times higher among the caregivers of the elderly people who had probable depression as per the total GDS scores (Adj OR: 6.83, CI: 3.04–15.32). Associated factors significantly affecting the family caregiver burden are summarized in Table 3.

### 3.6. Factors Affecting the Caregiver Time-Dependence Burden

We noted that those caregivers who were the main income supporter for their family had a 1.5 times higher time-dependence burden when compared to those who were not the main income supporter for their family (Adj OR: 1.52, CI: 1.09–2.12). Other significantly affected factors, such as female gender (Adj OR: 1.72, CI: 1.17–2.52), having diabetes (Adj OR: 4.09, CI: 2.34–7.16), caregivers of older persons with ADL total scores <12 (Adj OR: 7.06, CI: 2.80–17.81), and probable depression (Adj OR: 5.02, CI: 2.72–9.25), were similar to the factors affecting CBI ≥24, except for older-aged family caregivers and current smokers, as described in Table 4.

## 4. Discussion

This study explored the factors affecting the burden of family caregivers, both from the aspects of the care recipients’ demands and the concerns of the caregivers. In terms of the family caregiver burden, 5.5% had CBI total scores ≥24, indicating the need for some form of respite care to alleviate their burden. Similar to other studies, the time-dependence burden (25.7%), was the highest amongst the five subscales of the CBI, comprising time-dependence, physical, emotional, social, and developmental burdens. The level of caregiver burden in this study was lower than that of other studies reported in Southeast Asian countries such as Malaysia, Vietnam, and Thailand [22,23,24,25]. The subjective measures of the family caregiver burden as well as the interviewing of family caregivers together with their care recipients might have influenced the self-rated CBI reports. Moreover, the care recipients in this study were quite healthy requiring only modest assistance in their activities of daily living.

Older family caregivers were more likely to have a higher caregiver burden than those who were younger than 60 years of age, similar to other studies in Thailand and Europe [26,27,28]. The association between the caregiver’s physical health and stressors as they age could explain the increased burden among older family caregivers [29,30]. In terms of age difference, the mean age of caregivers and care recipients differed slightly (69.16 ± 8.33 years vs. 55.27 ± 13.7 years) showing that older adults were generally taken care of by same-generation caregivers. This may be explained by the reduction in family size and the migration of adult children for their job opportunities resulting in more couples taking care of each other at home. Two-thirds (67.6%) of caregivers of married care recipients were their spouses, further supporting this finding. Compared to younger caregivers, these same-generation caregivers would have more age-related physical and cognitive declines, themselves becoming older care recipients in the near future. Therefore, examining the factors affecting the caregiver burden is crucial when preparing for the sustainability of aging in place and promotion of healthy aging among senior citizens and their family caregivers. As in other studies, our study showed that aging care is mainly provided by females. Moreover, being female influences the extent of caregiver burden with several studies finding that female caregivers tend to report more health problems and symptoms of depression than male caregivers [23,26,31].Similarly, in our study we noted that female caregivers had a 2.5 times higher caregiver burden compared to male caregivers.

Regarding underlying diseases of caregivers affecting their burden, 9.3% of study participants were diabetic caregivers and having diabetes had a significant association with caregiver burden total scores of ≥24. This might be due to the added burden of managing their own diabetes along with taking care of their older care recipients. Another study in the European context also noted that caregivers with diabetes who were taking care of Alzheimer’s disease patients had increased odds of outpatient visits for their own health care compared to non-diabetic caregivers, although the caregiver diabetes status did not have any direct effects on the caregiver burden in that study [32]. However, caregiver diabetes in our study was positively associated with a higher caregiver burden, with diabetic caregivers having a 6.5 times higher burden in terms of CBI scores compared to non-diabetic caregivers. This finding should be addressed as the prevalence of non-communicable diseases, for example diabetes, has been accelerating in aging societies. This is due to common causes, such as obesity and sedentary lifestyles, amplified by rising life expectancy and the aging process affecting glucose metabolism and pancreatic islet dysfunction [33,34]. Moreover, there will also be an increased burden from disabilities related to diabetes, which in turn could lead to increases in the negative consequences of chronic diseases, the burden to family caregivers, and subsequent health care expenditures.

Health behaviors of the family caregivers, such as alcohol drinking and exercise habits, were not significantly associated with their caregiver burden. Nonetheless, the caregiver burden of current smokers was more than 4 times higher compared to non-smokers. This may be due to the fact that smoking is an important health behavior that has been associated with overcoming caregiver stress and burden [35]. Substance abuse may be the cause or effect, or both, of burden, as the poor health habits of burdened caregivers may be magnified increasing metabolic and vascular risk factors [36].

Our study population consisted of 3.3% of moderately to severely dependent care recipients. The family caregivers of such care recipients, with total ADL scores lower than 12, were 6.4 times more likely to have a higher burden than those taking care of care recipients with total ADL scores equal to or higher than 12. The findings of this study clearly show the negative consequences of poor functional ability of care recipients on the burden of family caregivers. Our study also showed that poorer health of care recipients also adversely affects the family caregiver’s burden, since the caregiver burden was higher among caregivers who were taking care of older participants with moderate to severe dependency. This is consistent with the findings from other studies [24,37,38,39]. As age increases, functional ability generally decreases and older people become more dependent on others, subsequently resulting in a higher burden on their caregivers. In addition to physical health, the mental health of care recipients also had significant effects on the extent of the caregiver burden. There was an association between the depression scores of care recipients and the burden of family caregivers, with the caregiver burden rising to nearly 7 times (6.86 times) higher when their care recipients had probable depression. Depression is a common mental health problem amongst Thai older people and, although the prevalence of 6.6% in this study population was lower than other studies in Thailand, the negative impacts of the older adult’s depression on the burden of family caregivers should be considered to help caregivers reduce their burden [40,41].

As the time-dependence domain was the highest among the five domains of the caregiver burden (25.7%), so factors affecting this domain were further explored. Being female caregivers, having diabetes, caregivers of seniors with moderate to severe dependency, and probable depression were significantly associated with time-dependence burden, whilst older age and current smokers did not have any correlations. Interestingly, an additional significant association between being the main income supporter of the family and time-dependence burden was noted. Since half of the family caregivers were the breadwinners of their families (49.9%), interference with work performance and reduced working hours due to caregiving could explain its relationship with the time-dependence burden. Moreover, difficulties within the workplace and poor career advancement may lead to a decline in the quality of care provided. These negative impacts of caregiving burden on the workforce vary according to national policies regarding caregiver support, such as the provision of respite care, day care, or financial support like care allowance or tax rebates. Countries with extensive long-term health care systems (e.g., Northern Europe) have reported fewer negative impacts compared to countries with lower level of supports (e.g., Southern Europe) [42,43].

There are some limitations in this study. First of all, we analyzed a baseline cross-sectional set of data and, therefore, causal interpretations of the results cannot be established. We can only suggest that the caregiver burden is associated with not only the care recipients’ functional ability and mental health, but also with the caregivers’ physical health, underlying diabetes, and health behaviors such as smoking. Secondly, the self-reported measures may lead to information bias, and subjective caregiver burden may also be underestimated by the concept of generalized reciprocity among family members, typical in Asian culture. Despite these limitations, our findings have useful implications for future interventions for the rapidly aging Thai community. Findings from our community survey reflect the current health status of both older adult care recipients and their caregivers, which may be useful in strengthening the existing services for aging care at home. Moreover, the association of diabetic caregivers with increased caregiver burden is an alarming feature that could have negative impacts on the family and subsequent increases in health expenditure for the country.

## 5. Conclusions

Although our study population mainly consisted of mildly dependent to independent older care recipients, the adverse effects of the care recipient’s demands and the caregivers’ profile on the family caregivers’ burden was clearly distinguished. The increasing burden of non-communicable diseases (NCDs) and their negative consequences on the caregiver burden, especially the finding of caregivers having diabetes or being current smokers being associated with a higher burden, highlights the need for health promotion activities targeting these modifiable risk factors. Such interventions will not only help to prevent NCDs, but also to reduce the burden of family caregivers who are the key players in the care of the aging population in Thailand. Despite the fact that the burden of family caregivers in this study was not so high, the local community should still consider implementing programs to educate caregivers and provide short-term respite care services to reduce or prevent caregiver burnout. National policies and interventions to assist families, such as respite care services, care capacity building, paid and unpaid leave for caregiving, etc., may reduce the burden of families struggling with informal care tasks. Furthermore, caregiver burden measurement can be applied as a screening tool to assess long-term care needs, complementing dependency assessment. Finally, implementation research is required to determine the effectiveness of respite care services for older people in order to strengthen and promote a sustainable traditional family-based long-term care system in Thailand.

## Figures and Tables

**Table 1:** Sociodemographic characteristics of the study participants, Maehia subdistrict, Chiang Mai, Thailand 2019.

Demographic Characteristics	Senior Citizens (*n* = 867)	Family Caregivers (*n* = 867)
	*n* (%)	*n* (%)
Age (mean ± SD)	69.16 ± 8.33	55.27 ± 13.7
<60 years		464 (53.5)
60–69 years	545 (62.9)	303 (34.9)
70–79 years	203 (23.4)	82 (9.5)
≥80 years	119 (13.7)	18 (2.1)
**Gender**		
Male	367 (42.3)	327 (37.7)
Female	500 (57.7)	540 (62.3)
Marital status		
Married	559 (64.5)	611 (70.5)
Single	52 (6.0)	183 (21.1)
Not currently married (separated, divorced, widowed)	256 (29.5)	73 (8.4)
**Education**		
Primary school	461 (53.2)	287 (33.1)
Secondary school and above	406 (46.8)	580 (66.9)
**Occupation**		
No job	629 (72.5)	270 (31.1)
Own business	54 (6.2)	253 (29.2)
Company/office staff	91 (10.5)	202 (23.3)
Daily labor	93 (10.7)	142 (16.4)
**Main Income Supporter of Family**		
No	564 (65.1)	434 (50.1)
Yes	303 (34.9)	433 (49.9)
**Underlying Diseases**		
**Hypertension**		
No	466 (53.7)	624 (72.0)
Yes	401 (46.3)	243 (28.0)
**Diabetes**		
No	716 (82.6)	786 (90.7)
Yes	151 (17.4)	81 (9.3)
**Hyperlipidemia**		
No	698 (80.5)	796 (91.8)
Yes	169 (19.5)	71 (8.2)
**Current Smoker**		
No	811 (93.5)	791 (91.2)
Yes	56 (6.5)	76 (8.8)
**Current Alcohol drinker**		
No	670 (77.3)	627 (72.3)
Yes	197 (22.7)	240 (27.7)
**Exercise**		
No Exercise	229 (26.4)	182 (21.0)
Exercise but not regular	472 (54.4)	569 (65.6)
Regular Exercise	166 (19.1)	116 (13.4)

**Table 2:** Care recipients’ demands and their family caregiver burden, Maehia subdistrict, Chiang Mai, Thailand, 2019.

Care Recipients’ Demands and Their Family Caregiver Burden	*n*	(%)
**Level of Dependency of Care Recipients According to ADL Total Scores**		
Mildly dependent to independent (≥12)	838	96.7
Moderately to severely dependent (0–11)	29	3.3
**Depression of Care Recipients According to GDS Total Scores**		
No depression (0–5)	810	93.4
Probable depression (6–15)	57	6.6
**Caregiver Burden of Family Caregivers According to CBI Total Scores**		
<24	819	94.5
≥24	48	5.5

ADL: Barthel’s Activities of Daily Living, GDS: 15-item Geriatric Depression Scale, CBI: 24-item Caregiver Burden Inventory Scale.

**Table 3:** Factors affecting the family caregiver burden, indicating the needs for respite care, Maehia subdistrict, Chiang Mai, Thailand, 2019.

Demography	Factors Affecting the Family Caregiver Burden Inventory Total Scores ≥24			
	Frequency (%)	Adjusted OR	95% Confidence Interval	
			Lower	Upper
** Age of Family Caregiver**				
<60 years	17(3.7)	Referent		
≥60 years	31(7.7)	2.14 *	1.03	4.44
** Gender of Family Caregiver**				
Male	13(4.0)	Referent		
Female	35(6.5)	2.25 *	1.02	4.94
** Caregiver being main Income Supporter of Family**				
No	20 (4.6)	Referent		
Yes	28 (6.5)	1.54	0.80	2.97
** Underlying Diseases of Family Caregiver**				
**Hypertension**				
No	32 (5.1)	Referent		
Yes	16 (6.6)	0.60	0.26	1.36
**Diabetes**				
No	34 (4.3)	Referent		
Yes	14 (17.3)	6.76 **	2.75	16.59
**Hyperlipidemia**				
No	43 (5.4)	Referent		
Yes	5 (7.0)	0.79	0.26	2.38
**Current Smoker**				
No	43 (5.4)	Referent		
Yes	5 (6.6)	4.31 *	1.30	14.28
**Current Alcohol Drinker**				
No	39 (6.2)	Referent		
Yes	9 (3.8)	0.49	0.19	1.25
** Total ADL Scores of Care Recipients**				
Mildly dependent to independent (≥12)	38 (4.5)	Referent		
Moderately to severely dependent (0–11)	10 (34.5)	6.36 **	2.37	17.06
** Total GDS Scores of Care Recipients**				
No depression (0–5)	33 (4.1)	Referent		
Probable depression (6–15)	15 (26.3)	6.83 **	3.04	15.32

ADL: Barthel’s Activities of Daily Living, GDS: 15-item Geriatric Depression Scale, Adjusted OR: Adjusted odds ratio, ** *p* value < 0.01, * *p* value < 0.05.

**Table 4:** Factors affecting the time-dependence burden of family caregivers, Maehia subdistrict, Chiang Mai, Thailand, 2019.

Demography	Factors Affecting the Caregiver Time Burden			
	Frequency (%)	Adjusted OR	95% Confidence Interval	
			Lower	Upper
** Age of Family Caregiver**				
<60 years	108 (23.3)	Referent		
≥60 years	115 (28.5)	1.13	0.78	1.63
** Gender of Family Caregiver**				
Male	71 (21.7)	Referent		
Female	152 (28.1)	1.72 *	1.17	2.52
** Caregiver being main Income Supporter of Family**				
No	98 (22.6)	Referent		
Yes	125 (28.9)	1.52 *	1.09	2.12
** Underlying Diseases of Family Caregiver**				
** Hypertension**				
No	150 (24.0)	Referent		
Yes	73 (30.0)	1.02	0.66	1.56
** Diabetes**				
No	181 (23.0)	Referent		
Yes	42 (51.9)	4.09 **	2.34	7.16
** Hyperlipidemia**				
No	200 (25.1)	Referent		
Yes	23 (32.4)	0.99	0.54	1.80
** Current Smoker**				
No	206 (26.0)	Referent		
Yes	17 (22.4)	1.40	0.73	2.71
** Current Alcohol Drinker**				
No	171 (27.3)	Referent		
Yes	52 (21.7)	0.79	0.52	1.20
** Total ADL Scores of Care Recipients**				
Mildly dependent to independent (≥12)	201 (24.0)	Referent		
Moderately to severely dependent (0–11)	22 (75.9)	7.06 **	2.80	17.81
** Total GDS Scores of Care Recipients**				
No depression (0–5)	187 (23.1)	Referent		
Probable depression (6–15)	36 (63.2)	5.02 **	2.72	9.25

ADL: Barthel’s Activities of Daily Living, GDS: 15-item Geriatric Depression Scale, Adjusted OR: Adjusted odds ratio, ** *p* value < 0.01, * *p* value < 0.05.

## Data Availability

The data presented in this study are available on request from the corresponding author. The data are not publicly available because this study was a subgroup analysis of baseline data of the intervention clusters from a cluster randomized trial and the final analysis was ongoing, and publications of the whole cluster randomized trial have not been finished yet.
